# Pharmacological Neuroenhancement: Current Aspects of Categorization, Epidemiology, Pharmacology, Drug Development, Ethics, and Future Perspectives

**DOI:** 10.1155/2021/8823383

**Published:** 2021-01-13

**Authors:** Johanna Daubner, Muhammad Imran Arshaad, Christina Henseler, Jürgen Hescheler, Dan Ehninger, Karl Broich, Oliver Rawashdeh, Anna Papazoglou, Marco Weiergräber

**Affiliations:** ^1^Experimental Neuropsychopharmacology, Federal Institute for Drugs and Medical Devices (Bundesinstitut für Arzneimittel und Medizinprodukte, BfArM), Kurt-Georg-Kiesinger-Allee 3, 53175 Bonn, Germany; ^2^Institute of Neurophysiology, University of Cologne, Faculty of Medicine, Robert-Koch-Str. 39, 50931 Cologne, Germany; ^3^Molecular and Cellular Cognition, German Center for Neurodegenerative Diseases (Deutsches Zentrum für Neurodegenerative Erkrankungen, DZNE), Sigmund-Freud-Str. 27, 53127 Bonn, Germany; ^4^Federal Institute for Drugs and Medical Devices (Bundesinstitut für Arzneimittel und Medizinprodukte, BfArM), Kurt-Georg-Kiesinger-Allee 3, 53175 Bonn, Germany; ^5^School of Biomedical Sciences, Faculty of Medicine, University of Queensland, Brisbane, Australia

## Abstract

Recent pharmacoepidemiologic studies suggest that pharmacological neuroenhancement (pNE) and mood enhancement are globally expanding phenomena with distinctly different regional characteristics. Sociocultural and regulatory aspects, as well as health policies, play a central role in addition to medical care and prescription practices. The users mainly display self-involved motivations related to cognitive enhancement, emotional stability, and adaptivity. Natural stimulants, as well as drugs, represent substance abuse groups. The latter comprise purines, methylxanthines, phenylethylamines, modafinil, nootropics, antidepressants but also benzodiazepines, *β*-adrenoceptor antagonists, and cannabis. Predominant pharmacodynamic target structures of these substances are the noradrenergic/dopaminergic and cholinergic receptor/transporter systems. Further targets comprise adenosine, serotonin, and glutamate receptors. Meta-analyses of randomized-controlled studies in healthy individuals show no or very limited verifiability of positive effects of pNE on attention, vigilance, learning, and memory. Only some members of the substance abuse groups, i.e., phenylethylamines and modafinil, display positive effects on attention and vigilance that are comparable to caffeinated drinks. However, the development of new antidementia drugs will increase the availability and the potential abuse of pNE. Social education, restrictive regulatory measures, and consistent medical prescription practices are essential to restrict the phenomenon of neuroenhancement with its social, medical, and ethical implications. This review provides a comprehensive overview of the highly dynamic field of pharmacological neuroenhancement and elaborates the dramatic challenges for the medical, sociocultural, and ethical fundaments of society.

## 1. Definition and Social Implications of Neuroenhancemen

### 1.1. General Aspects

**Figure 1:**
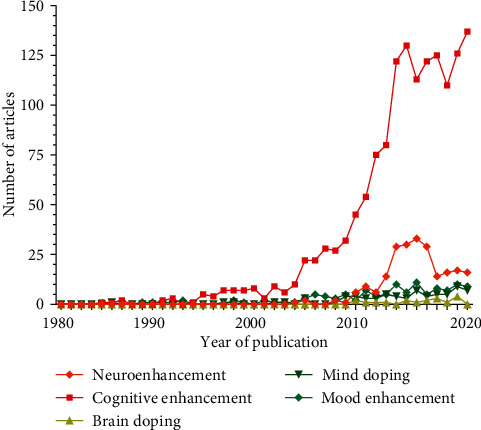
Annual number of publications in the field of pharmacological neuroenhancement. The results of a PubMed literature search using the individual keywords “neuroenhancement,” “cognitive enhancement,” “mood enhancement,” “mind doping,” and “brain doping” are displayed from 1980 to 2020 (1527 publications in total). Note that there is a nearly exponential increase in the overall publication rate till 2013 which became stable within the last years.

Neuroenhancement or “brain-doping” has gained increasing social and media attention and has dominated scientific research in the field in recent years [1–3]. Different, partly popular scientific terminology and paraphrases such as *neuroenhancement*, *neuropusher*, *brain booster*, *mind doping*, *smart drugs*, *viagra for the brain*, *botox for the brain*, *cognitive enhancement*, *brain doping*, *arms race in the head*, *nootropics*, *cosmetic neurology*, *cosmetic pharmacology*, and *psychopharmaceuticalization of society* demonstrate a far-reaching conceptual blurring (Figure 1). For a scientifically based approach to the phenomenon of neuroenhancement, a consensus definition is indispensable [4–6]. As a core definition, pharmacological neuroenhancement (pNE) implies the attempt of healthy individuals to augment attention and vigilance, learning and memory, mood, and behavior, among others, by taking prescription drugs. Furthermore, intake of pNE drugs is neither medically indicated (primary prevention) nor is it reasonable as secondary prevention in healthy individuals [7–10]. Thus, the pharmacologically active compounds used for pNE are not supposed to be medically prescribed, and consumption is not for reasons of pleasure [6, 11]. The enjoyment and/or enhancement character of pNE drugs is defined by teleology, motivation, and dosage, which is of special relevance for the disputable pNE categorization of nonprescription drugs and/or herbal ingredients, such as caffeine (in coffee, energy drinks, etc.) and ginkgo biloba extracts, but also food supplements, nicotine, and alcohol. In addition, illicit soft and hard drugs, e.g., amphetamines, lysergic acid diethylamide (LSD), heroin, cocaine, 3-methylmethcathinone (3MMC), psychedelic mushrooms, synthetic cannabinoids, and dimethyltryptamine (DMT), might also be used for pNE. Thus, gray areas exist in the definition of pNE, since a clear distinction between enjoyment and enhancement is only partially possible due to their conceptual and content blurring [12, 13]. It should be mentioned that other definitions of pNE also exist which include usage in people suffering from illnesses [14]. However, as legal aspects are of central relevance in pNE drug abuse, our primary focus is on prescription drugs used in individuals without a related diagnosable ICD (International Classification of Diseases) or DSM-5 (Diagnostic and Statistical Manual of Mental Disorders 5) defined condition.

**Figure 2:**
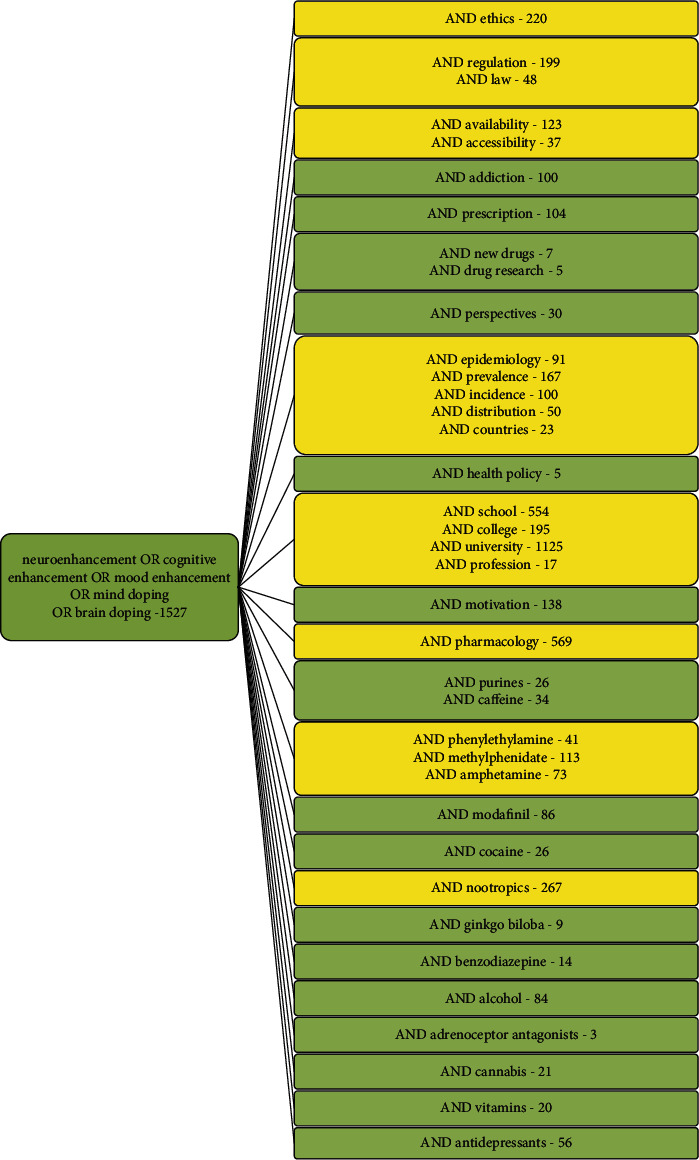
Number of publications in pNE and related subfields. Results of a PubMed literature search using relevant keywords for pNE and pNE subtopics. Publications till 11/2020 are considered in this query. Based on the number of articles, some aspects, such as epidemiology, accessibility and diversion strategies, pharmacology, and ethics, seem to attract specific scientific attention (highlighted in yellow).

Notably, pNE is a highly controversial topic in public health discussions, and an increasing number of scientific publications are dealing with pNE and related topics. The overall annual number of articles in the field displays exponential characteristics since 2000 with a flattening tendency in recent years (Figure 1). A more detailed quantitative publication analysis further reveals that some pNE aspects are more strongly represented in literature, such as epidemiology, accessibility and pNE distribution patterns, pharmacology, and ethics, for example (Figure 2).

The controversy of pNE mainly results from changing definitions of health and disease, the gradual transition from natural stimulants to pharmacotherapeutics used for pNE, differences in the interpretation of benefit/risk aspects, and different attitudes regarding ethical concerns. This review will first elaborate the concept of health and disease, as this is fundamental for the pharmacological, legal, regulatory, and ethical status of pNE. We then concentrate on the different motivations for pNE and potential expectations and concerns of users. Afterwards, we show pharmacoepidemiological data about the worldwide use of pNE, and how pNE drugs are acquired by users. We finally present pharmacodynamic, pharmacokinetic, risk and efficacy data on the major substance groups utilized as pNE drugs, and comment on recent advances in drug research and development and potential candidates “in the pipeline” that might be misused/abused for pNE in the future. This aspect is integrated into the final consideration of ethics in pNE.

### 1.2. The Concept of Health and Disease and Its Relevance for pNE

The current World Health Organization (WHO) definition formulated in 1946 defines health as a “state of complete physical, mental, and social well-being and not merely the absence of disease or infirmity” (https://www.who.int/about/who-we-are/constitution). It is widely noted that this definition no longer fits the current purposes of health policies. Instead, it has been suggested to change the emphasis towards the ability to adapt and self-manage in the face of physical, social, and emotional challenges [15]. Importantly, it overcame the negative, exclusive definition of health as the absence of disease and incorporated the relevance of physical, social, and mental domains.

More than 70 years later, criticism of the original definition of health is on the rise due to significant changes in demography, disease patterns, and public health care systems, which render the original definition counterproductive [15–19].

A central criticism of the WHO's health definition is related to the absoluteness of the term “complete” concerning well-being, which contributes to an unintentional medicalization of society. The absolutism of well-being is indeed largely “impracticable” because “complete” is neither operational nor measurable, and even an optimist would have to accept the impossibility of risk-free well-being [16, 19]. Thus, health is hardly a “state of complete physical, social, and mental well-being” nor is it “merely the absence of disease or infirmity”. It was supposed to provide a transformative view of “health for all,” which went beyond the prevailing negative conception of health based on an “absence” of pathology.

The struggle to achieve complete health “would indicate that most of us are unhealthy most of the time,” which is of high relevance for the interpretation of pNE [19]. This approach generally supports the current tendencies of academic institutions, drug research and development industries, and other stakeholders to redefine “diseases” and to expand the general scope of healthcare systems. As a consequence, this interpretation can also lead to the detection of abnormalities or deficiencies at levels that might never cause or be an illness. Further, pharmaceutical companies produce drugs for “conditions” that have not been previously defined as health issues, or these drugs are used off-label or are abused/misused not to restore a pathological state but to improve further or optimize a physiological state as holds true for pNE. The considerable changes in the demography of populations in the last decades and the nature of chronic neuropsychiatric and neurodegenerative diseases, for example, have enhanced related drugresearch and development and aggravated the phenomenon of pNE. The number of people living with chronic neurodegenerative diseases such as Alzheimer's disease (AD) is increasing worldwide for decades. Thus, aging with chronic illnesses has become the norm, and chronic diseases, such as AD, account for most of the expenditures of the healthcare system. Based on the WHO definition, individuals suffering from chronic diseases and disabilities, but also those who feel unpleasant or suboptimal in their performance, are termed ill. This view, including aspects of quality of life, largely ignores the role of the human capacity to cope with ever-changing physical, emotional, and social challenges and to function with fulfillment and a feeling of well-being either with a chronic disease/disability or cognitive challenges as in pNE.

It should also be considered that the definition of health severely affects health policies and is affected by cultural aspects and future scientific and technological advances. Thus, the interpretations of health are highly context-dependent, as human disease states only exist in relation to people in varied cultural environments. Results from medical anthropology and sociology have illustrated that whether people believe themselves to be ill, varies with gender, social class/economics, ethnic group, proximity to family support, historical time, and the associated increasing expectations of health-related improvements in diagnostics, but mostly for a mixture of social and economic reasons [20]. Thus, health definitions have an impact on prevention programs, health care issues, and the determination of outcome measures. Consequently, the definition of health has to meet the demands of practical life and measurement purposes. Importantly, there is a strong effort to shift the current static formulation towards a more dynamic one, prioritizing social skills and the resilience or capacity to cope, maintain, and restore individual integrity, equilibrium, and sense of well-being rather than improved survival rates and complete recovery [15, 20]. Alternative concepts of health that have been proposed, provide more emphasis on the ability to adapt and to self-manage in the three domains of health (physical, mental, and social) [21–24].

To discriminate health, it is worth having a look at the definition of disease too. A standard definition states that disease is “a definite pathological process having a characteristic set of signs and symptoms. It may affect the whole body or any of its parts, and its etiology, pathology, and prognosis may be known or unknown” [25]. Another definition was suggested by Campbell et al. (1979): “in medical discourse, the name of a disease refers to the sum of the abnormal phenomena displayed by a group of living organisms in association with a specified common characteristic or set of characteristics by which they differ from the norm of their species in such a way as to place them at a biological disadvantage.” [26]. Disease can refer to a combination of signs and symptoms, phenomena associated with a disorder of function or structure, or illness associated with a specific cause. There are, however, no universally accepted criteria for defining “disease” [27].

In summary, it is generally accepted that pNE is used by healthy individuals, although we are aware that the term “health” is subject to wide-spread interpretations. On the other hand, drugs used for pNE are designated to treat defined disease states, and motivating factors for pNE, as outlined below, do not meet these criteria.

### 1.3. Nonpharmacological NE

In addition to the pNE mentioned above, there are further enhancement strategies that are based on surgical, neurotechnological methods (low-frequency repetitive transcranial magnetic stimulation (LF-rTMS), transcranial direct current stimulation (tDCS), deep brain stimulation with depth electrodes, and CNS interface) or behavioral approaches [12]. While surgical and neuro/magnetic stimulatory enhancement was mostly experimental or fictive in the past, it has become increasingly feasible in recent years, particularly in association with brain-computer interface technology or the man-machine hybrid (cybernetic organism).

Media presentation often dramatizes real conditions, especially on pNE [28]. For example, pNE was characterized as a habitual procedure among healthy students and ranked as an alarm signal [29]. In some cases, scientific evidence for such an increased usage of pNE is still limited. Unfortunately, inadequate and inconsistent definitions of pNE have impaired its significance and prevalence evaluations in many large-scale surveys and cross-cultural considerations [30].

For example, lifetime prevalence data for the nonmedically indicated use of US prescription stimulants vary from 5% to 35% [31, 32], without determining the specific personal goals and expectations of the application. Surveys from Germany suggest a lower prevalence, ranging from 0.8% [4] to 2.0%, e.g., for university students [33]. Detailed epidemiological studies considering the motivational background for pNE are thus mandatory and presented below.

## 2. Motivational Basics for pNE

The main motivations for healthy individuals to apply pNE can be systematically categorized as follows [12]:

- so-called cognitive neuroenhancement: the enhancement of mental performance parameters in the field of attention, vigilance, learning and memory, as well as concentration, is in the foreground
- so-called emotional neuroenhancement (mood enhancement): an optimized mood and/or a modulation of personality traits such as social skills are in the focus of this approach. Users also anticipate an accompanying positive effect on cognitive parameters
- so-called moral neuroenhancement and negative neuroenhancement: moral enhancement is gaining increasing attention. In the trauma therapeutic field, the inhibition of consolidation of new potentially traumatic memory engrams, as well as the extinction of existing traumatic memories, is a promising field of application. Also, empathic limitation (empathic fatigue) due to physical and/or mental exhaustion is a potential application field. As with cognitive and emotional enhancement, the dangers of direct and indirect moral enhancement are manifold, including limitation of freedom and autonomy, failure, and moral degradation [34].

We are currently facing a highly competitive working environment and demanding culture in combination with the idealization of a fulfilled private reality. This requires an outstanding, enduring motivation and performance at the cognitive level as well as emotional stability, particularly under stressful situations. In professional life, omnipresent evaluation and rating processes, which seem to make human productivity transparent in a simple way, strongly contribute to these real and/or perceived stress scenarios. Moreover, there are sociomedia indoctrinated expectations and idealization processes to a seemingly fulfilled professional and family life. Thus, the maximum development of possible talents and abilities, which are not limited by individual physical and mental deficiencies, turns out to be the highest maxim to act upon.

A central, novel motivational aspect in pNE is related to the increasing demands of the digital society, where information technology has resulted in fundamental societal shifts. It led to substantially new organizing principles for private agents and public institutions and has fundamentally rearranged the private and public sector [35].

Information technology became the Holy Grail in information transfer, storage, and sharing in the multidimensional digital society. Individual skills are now focused on keeping pace with knowledge development and knowledge management in terms of access, processing, and interpretation in the omnipresent online environment [35]. This knowledge is infrastructured in a dynamic tentative national or global online architecture that is accessible to individual users via various search tools and engines and related operative algorithms. All these groundbreaking alterations in our digital society request for exceptional novel personal skills in qualified, critical, and continuous decision-making in an all-encompassing ever-changing knowledge fluid [36–38]. In contrast, this development undermines and questions traditional past patterns of reminiscence, leaving individuals in an atmosphere of reduced secureness and degree of reliance.

Altogether, current society is the most sophisticated, high-tech, and high-cost with the permanent need to learn and update skills and knowledge to be able to manage one's own lives. As digital society is based on software that is continuously updated, digital navigation skills are a hallmark for navigating through life, and knowledge became a crucial quality in current society. For an increasing number of individuals, pNE, therefore, represents a potential escape strategy from this scenario of sociocultural oppressiveness [1, 39–43].

The application patterns of pNE do not seem to differ significantly between men and women. Besides classical oral uptake, other, non-oral routes of application were also described, such as injections and intranasal administration, with the latter being more common than injections [44–47]. These para-oral routes of administration often seem to be associated with the use of other illicit drugs, e.g., ecstasy, cocaine, cannabis, and amphetamine [48, 49]. While differences in application patterns between men and women are not evident, there are gender-specific differences in motivation [50]. Improved performance in studies, occupation, and social interaction is often mentioned, as well as an improvement in concentration and stamina, reduction of fatigue, but also the induction of an ecstasy-like state, reduction of boredom, coping strategies, reduction of emotional stress (distress), improvement of relaxation, increased sexual performance, and weight loss. Women predominantly apply pNE for learning support to strengthen their stamina and to reduce fatigue, stress, and weight. In men, however, the induction of an ecstasy-like state and a supposedly better sexual performance play a decisive role [50].

Many of the original studies were carried out in college students and revealed that academic motivations play a major role. In most surveys, for example, 50-89% of college students reported that academic reasons were the most common motivations [45, 47, 51–70]. The overall aim to be more productive was cited by 40% of adults reporting nonmedical use in a US survey [71]. The desire to improve academic and/or work performance was named by 38-57% of the US adults who admitted nonmedical use of stimulants [72]. Importantly, recreation turned out to be the second most commonly cited motivation, and “getting high” was a central motivation for nonmedical use of stimulants by 2-31% of college students [47, 51–53, 55, 57, 60, 62, 73, 74]. Another survey of 4,580 college students elicited that 31% of nonmedical users of psychostimulants tried to “get high.” Notably, there are region and race-specific differences. For African-American students, for example, “getting high” was not a predominant motivation [60]. Besides academic/work improvement and recreation, curiosity is another relevant motivation. Among college students, 17-31% stated that they were curious or simply wanted to experiment with psychostimulants [51, 53, 54, 60, 61]. Further motivations among college students include the enhancement of alcohol effects, enhancing social situations, and improving socialization [51, 56, 62, 63]. Enhancing stamina while partying with friends turned out to be another important motivation for nonmedical use of stimulants among college students [45, 51, 53–59]. Astonishingly, 12% [52] and 4% [57] of the US college students reported misuse of psychostimulants due to a self-medicated and undiagnosed ADHD. In parents with suspected or diagnosed ADHD, there was a 2.6 times higher chance to use or to have used their child's stimulants [75]. Finally, weight loss also turned out to be a motivation for nonmedical use, with rates among the US college populations ranging from 3.5 to 11.7% [51, 52, 76–78]. Among the US women in the college age who either suffered from an eating disorder or were at risk for it, 17% stated that controlling of body shape and body weight were major motivations for use of psychostimulant [79]. Notably, many students have disclosed the use of pNEs to their parents, and the latter were reported to facilitate or encourage their children to continue using them, often with the intention to improve their academic outcome [80]. It cannot be excluded that some parents urge or even force their children to use pNE as they financially invest in the academic/professional success of their children. Up to now, there are no common, accepted rules/measures that control or forbid the use of pNE in an academic setting.

In addition to the exclusively self-related motivations outlined above, altruistic motives for pNE are also relevant. The latter applies to specific occupational groups such as soldiers and physicians, in rare emergencies, such as pandemics. From ethical standards, these fields of application require a differentiated consideration.

## 3. Positive Expectations and Critical Reflection of pNE

Positive and negative expectations are of specific relevance in the field of pNE. According to a national report of a German health insurance company from 2009, 25% of respondents consider brain doping as an acceptable approach [81]. While 50% still fear that the risks of pNE intake could outweigh the benefits, i.e., negative benefit/risk ratio, 20% estimate the benefits higher than the risks, i.e., positive benefit/risk ratio. About 60% of respondents categorically refuse brain doping, but only 3% due to ethical reasons, i.e., because of an undeserved advantage. Despite a substantial increase in application rates (see below), many individuals are concerned about the safety of pNE application. In the EU study, NERRI (Neuroenhancement—Responsible Research and Innovation 2013-2016), 76% of participants stated that pNE should not be used in children/adolescents, and 74% remarked that there should be regulatory control and monitoring of pNE [14]. Interestingly, 69% and 66% of the respondents, respectively, favored the appreciation of individual achievements without pNE support and suggested a decrease in working occupational burden [14]. Paradoxically, however, 68% of study participants interpret pNE usage as an intrinsic feature of human nature to overcome the physiological limitations of the central nervous system (CNS) and 71% to cope with the increasing demands of life. Furthermore, 43% believe that from a pharmacovigilance perspective, a “safe” neuroenhancer should be available as a consumer product [14]. These statistics demonstrate the ambivalent relationship of respondents to brain doping. Many individuals seem to feel compelled to pNE by their professional and private reality of life, although their general attitude to pNE is negative. In Germany, about 83% of the working population reject pNE [82]. Notably, 21.6% of users report that, e.g., psychostimulants did not meet their expectations, 50.4% were satisfied, and in about 25%, pNE exceeded their expectations [50]. From a regulatory point of view, it should be noted that the benefit/risk ratio for pNE is always negative by definition, as each psychostimulant has more or less dominant side effects, but is medically not indicated in healthy individuals. Therefore, the usage of pNE cannot have a disease-related benefit from a regulatory or drug law perspective.

## 4. Pharmacoepidemiological Characteristics of pNE

The practicing clientele of pNE typically comprises individuals with high intellectual and/or emotional/social demand for performance, which is often intrinsically motivated but may also be extrinsically imposed. pNE users and susceptible groups primarily involve students and employees with predominantly cognitive activity conducting either sporadic situational or habitual pNE. In addition, professions with predominant night work, shift, or extended work are also affected, as pNE is supposed to attenuate the effects of sleep deprivation and increasing workloads [83]. Notably, the misuse of pNE drugs can also increase the susceptibility to use alcohol and illicit substances [44, 47, 49, 84–90].

In 2009, a German health insurance company presented results of a survey of 20,000 employees aged 20-50 years, which demonstrated that 5% of respondents had taken “drugs for brain doping” at least once in the past; 50% indicated to carry out pNE to improve mood and 43% to counteract anxiety symptoms [81]. Importantly, 2% of all respondents admitted regular brain doping, resulting in an extrapolated total number of 800,000-2,000,000 pNE users in Germany. Additional subsequent evaluation in 2015 exhibited an increased prevalence of pNE compared to the 2009 survey. Almost 7% of respondents stated that they had utilized pNE once in their lifetime. Including the estimated number of unreported cases, however, a total fraction of 12% is more likely. Furthermore, the survey suggested that the fraction of current users (at the time point of evaluation) was 3% and up to 6% including the estimated number of unreported cases [82]. Two percent of the survey respondents confirmed habitual consumption of pNE related drugs. Including the estimated number of unreported cases, the number increases up to 3.5% [82].

A 2008 survey in 60 countries by the Journal Nature revealed that 20% of respondents had already tried pNE, 80% endorsed brain doping, and 34% had received pNE medication from the internet, 14% from a pharmacy, and 52% from a physician. A US study from 2006 further ascertained that 8.1% of students had taken prescription drugs for pNE once in their lifetime and 5.4% within the last year [42]. In a German survey among 1,000 students and 500 pupils, 1-2% had carried out brain doping at least once, 10% categorically refused brain doping, and 80% requested as a prerequisite that brain doping does not cause side effects, long-term damage, and addiction [6]. Further surveys demonstrated that 17-25% of the US students use psychostimulants [42], 7-25% of Americans regularly take antidepressants, and 20 million Americans regularly consume fluoxetine [5, 6, 91–93]. Additional national surveys include the “HISBUS” study [94, 95] and the “Kolibri study” of the Robert Koch Institute (Germany) [96].

A recent survey in Germany from 2018 revealed pNE prevalences of 4.3% for prescription drugs without medical indication, 10.2% for illicit drugs, 20.3% for mood enhancers, and 23.4% for cannabis [7]. In this study, it became apparent that the use of pNE is also associated with reduced psychological resilience [7].

In the university educational system, not all faculties seem to be equally affected by pNE. An Austrian study ascertained that students of technical courses and computer sciences are the least affected by pNE (5.4%). The percentage of users increased in natural sciences (11.5%), social sciences (11.5%), economics and law (12.5%), and finally culminated in the field of medicine, pharmacy, and psychology with 14,9% [8].

The web-based Global Drug Survey (GDS) from 2015 and 2017 investigated pNE practices in various countries throughout the world [50, 97]. The GDS is of particular relevance as it collects numerous parameters. These include sociocultural differences (country-specific economics, education systems, performance society, etc.) and differences in health care and national regulations (legal and illegal access to medicines, prescriptive practices, restrictive or permissive handling of stimulants/drugs, etc.). It also suggests how these factors could influence and/or aggravate the spread of pNE.

For the nonprescriptive use of psychostimulants such as methylphenidate, or dexamphetamine, the GDS 2015-2017 demonstrates that pNE with prescription stimulants has the highest prevalence in the US (18.7% and 21.6%, respectively), followed by Canada (8.7% and 12.5%, respectively), the Netherlands (8.2% and 13.5%, respectively), and Belgium (3.6% and 12.4%, respectively). Furthermore, a dramatic increase in pNE was observed within the two-year evaluation period. In Germany, the statistics are still comparatively low ranging from 1.5% to 3.0%, respectively. The lowest prevalence was observed in Austria with 0.8% and 2.3%, respectively [50, 97]. An interesting phenomenon was observed in Iceland for which earlier evaluations from 2010 to 2011 revealed a steep increase in methylphenidate use. The per capita prescription rates exceeded those in the US by that time and turned out to be the highest prescription rate per capita in the world [44, 98]. As outlined below, national specificities contribute to these findings.

The nonprescriptive misuse of modafinil for pNE 2015-2017 is dominated by the UK (3.2% and 10.0%, respectively), Australia (2.1% and 5.5%, respectively), and Canada (0,8% and 2.7%, respectively). In the US, modafinil is common for pNE (1.4% and 4.1%, respectively), as well as in Germany (0.2% and 1.1%, respectively). However, with very few exceptions, prevalences are rising significantly in almost all countries surveyed. With 0.1% and 1.0% application rates for modafinil, Austria again exhibits the lowest prevalences in GDS statistics [50].

For illegal psychostimulants such as cocaine, amphetamine, and methamphetamine, the GDS 2015-2017 reveals high application rates in the US (4.4% and 14.7%, respectively), Canada (3.3% and 12.4%, respectively), the UK (1.9% and 13.3%, respectively), France (2.3% and 12.7%), the Netherlands (4.2% and 14.1%, respectively), Belgium (2.7% and 10.8%, respectively), and Hungary (2.8% and 10.7%, respectively). New Zealand (0.7% and 3.3%, respectively), Portugal (1.0% and 5.8%, respectively), and the German-speaking countries (Germany, (1.7% and 5.5%, respectively), Austria (1.5% and 6.7%, respectively), and Switzerland (1.4% and 5.9%, respectively)) exhibit the lowest prevalences [50, 97].

In summary, the GDS suggests a global prevalence of pNE using legal and illegal drugs of 4.9% in 2015. In 2017, the global prevalence had already risen by 180% to 13.7% and by more than 300% in “pNE hot spot countries”.

Interestingly, about 25% of pNE applicants in the GDS survey stated that they would reduce drug intake for pNE in the following year, but less than 3% would seek for professional help. Two-thirds of pNE users report habitual handling with up to 10 applications per year, whereas sporadic intake of psychostimulants rarely occurred. High-frequency applications (>100 applications/year) were typically observed in men (13.5%), whereas women are less affected (4.4%) [50, 97]. Most users were reported to apply pNE for a period of 1-2 weeks, e.g., for examinations or to compensate workload peaks 1-2 times a year [50].

## 5. Availability/Accessibility of pNE

Some of the drugs used for pNE such as methylphenidate and dexamphetamine (see below) are internationally classified as controlled drugs in many countries and classified as the highest category of abuse/dependence risk that can be assigned to a drug of medical benefit [99]. Given this potential for pharmaceutical stimulants to be abused, one of the major concerns about the prescription of these drugs is the risk of diversion and misuse. Per definition, diversion characterizes the transfer of medication from the person for whom it is prescribed to a person for whom it is not prescribed [44]. Diversion is in close proximity to misuse as the latter includes the use of stimulants not prescribed to the individual, but also the use of related medications in ways other than actually prescribed, e.g., via application of increased dosages and/or higher frequency intake [44]. Detailed national information on diversion and misuse of pharmaceutical stimulants from different regions in the world are limited [44]. To date, a major evidence for pharmaceutical stimulant misuse/diversion arises from studies of student populations in North America and Europe [32, 100]. Of the 21 studies identified by Wilens et al. (2008) [32], 71% were based on school and college students and diversion strategies predominately seem to occur here. Students are very much aware of related practices. For example, Weyandt et al. (2009) [65] reported that 7.5% of college students claimed use of psychostimulants within the past 30 days, whereas 60% were aware of other students who misused/distributed such stimulants. At least half of the study respondents confirmed that psychostimulants are easy to acquire on the campus [57, 65, 101]. Our specific knowledge on the prevalence of diversion, its potential regional specificities, and its underlying strategies is limited. Life-time data on diversion range from 11 to 29% in some publications [32, 88, 102–106]. Recently, however, even higher rates of 17–62% have been reported [46, 47, 107, 108]. Other studies have proposed a diversion prevalence for students with prescriptions being asked to sell, trade, or give away their medication, in the range of 23-84% [32, 44]. A major source (50–90%) for diversion was reported to be friends, peers, and family members [51]. Besides, many individuals (4–35%) reported nonmedical use of their own prescription stimulant [51]. It turned out that personal experience of diversion seems to be far less prevalent than the knowledge of diversion among others [32]. As an example, only 8% of students admitted to having sold or given away medication themselves. In contrast, 63% were aware of someone else who had distributed, e.g., psychostimulants, and 67% were aware of others who had used such stimulants for recreational purposes [101].

Various risk factors for diversion and misuse were described in different studies. The latter include being male, Caucasian, college student, suffering from a conduct and/or substance use disorder, using immediate-release drug formulations, and being a fraternity/sorority member [44, 58, 109, 110]. Importantly, gender differences have not always been detected consistently [85, 109, 111]. This risk factor analysis however might be biased, as related research was predominantly carried out in college students [44].

A survey among German employees who perform pNE revealed that 53.8% acquired drugs for pNE via a regular medical prescription, whereas 22.4% obtained drugs from a pharmacy without prescription [82]. These acquisition patterns can also be observed in other countries. Some individuals feign ADHD symptoms to receive a prescription from their health care provider [51]. Furthermore, 20% of the US adults who admitted past-year nonmedical use stated that they got prescriptions fraudulently from a doctor [51]. In general, these results are irritating, as most pNE-related drugs are prescription-only and/or listed as narcotic substances, and therefore should not occur at all in *lege artis* prescriptions for healthy individuals. Consequently, pNE applicants either purport a fictive medical need to the attending physician or directly consult their doctors for prescription of pharmacological neuroenhancers. The latter was reported by 40.8% of general practitioners [112]. It should be stated again that from a prescriptive point of view, these cases should not exist, and doctors should not service these accessibility strategies.

Another essential acquisition path is so-called e-commerce [6]. Whereas diversion of prescription medicines to third parties makes up, e.g., 14.1%, e-commerce accounts for only 8.5% of pNE cases in Germany [82]. However, the EAASM (European Alliance for Access of Safe Medicines, http://www.eaasm.eu) stated in 2008 that 90% of internet pharmacies offer prescription drug delivery without a prescription (formally not available, e.g., at German online pharmacies). The US National Center on Addiction and Substance Abuse (CASA, http://www.casacolumbia.org) stated in 2008 that 85% of internet pharmacies ignore fundamental prescription requirements. These pharmacies are illicit online pharmacies (IOPs) in contrast to legitimate online pharmacies (LOPs). Online pharmacies are a huge economic factor and have dramatically grown recently [113]. The market has increased at an annual rate of 17.7% with US $29.35 billion in 2014 and an anticipated global volume of US $128 billion in 2023 [113, 114]. The motivation to order from online pharmacies is mainly based on lower prices [115, 116], convenience, and the possibility to access drugs that are unavailable or difficult to obtain [113, 117, 118]. However, most users are not aware of the high percentage of IOPs which is in the range of 67-75% of web-based drug merchants [119]. This development is critical, as it opens the doors to uncontrolled access to prescription and/or controlled drugs and undermines professional and regulatory measures to guarantee high qualitative drug standards, drug supply chains, and the health of patients [113, 120, 121].

In general, individuals are advised to acquire medicines only from online retailers registered with the national competent authorities (e.g., in the EU Member States). This reduces the risk of buying medicines that are substandard or falsified. If pNE drugs are acquired via nonregistered online pharmacies without prescription, there are major safety concerns. The latter may contain ingredients of low quality with toxic impurities and wrong doses. They may be deliberately and fraudulently mislabeled as regards identity and source. Fake packaging and wrong or low levels of ingredients might further trigger misuse of the drugs. Notably, there is a black market with substances of highly questionable composition, pharmaceutical quality/purity with no legal and controlled production. In addition, such drugs may be harmful with unexpected and/or severe side effects and may exert potentially dangerous interactions with other medicines the patient is already taking.

Compared to the USA, the prescription regulations in Germany seem to be less undermined by diversion strategies and e-commerce than by nonindicated medical prescriptions.

Internationally, the GDS portraits a different picture: diversion strategies, e.g., via relatives and friends, account for 47.8% of pNE cases. In 11.8%, pNE drugs were acquired from a dealer; in 9.1%, drugs were obtained from the internet. Family members with prescriptions appeared in 6.1% for procurement, physicians only in 3.8%. It should also be noted that, in 25%, the physicians involved did not inform applicants about potential risks and side effects of pNE-related drugs [50].

## 6. Substance Groups

The fundamental physiological basics of attention and vigilance and learning and memory, as well as emotions, are discussed in detail elsewhere. Below, the most important classes of pNE drugs are characterized in terms of pharmacodynamic properties, effectiveness as potential neuroenhancers, and their side-effect profile (see also Table 1). Concerning national regulatory peculiarities, reference should be made to the corresponding documentation of the national/international regulatory authorities. It should be noted that the drug classes listed below primarily represent drugs with intentional use for pNE. They are a common subject of pNE research and pNE related surveys [50, 97].

### 6.1. Purines and Methylxanthine Derivatives

The best-known methylxanthines include caffeine, theophylline, and theobromine as part of many natural stimulants, as well as other derivatives such as bromotheophylline, chlorotheophylline, aminophylline, dipropylcyclopentylxanthine, doxofylline, and paraxanthine. Among others, caffeine can be found in coffee beans (coffea arabica: 0.96–2%), leaves of black tea (thea sinensis, 3-5%), mate (lex paraguarensis: 1.63%), kola nuts (cola niida, 1-2%), cocoa beans (theobroma cacao: 0.05-0.36%), and guarana (paullinia species: approx. 10%). Theophylline is a central ingredient in tea leaves (0.1%). Theobromine makes up the main alkaloid of cocoa beans (1.5-3%). The level of caffeine is 30 mg/0.33 ltr. Coca Cola, about 60-150 mg/cup of coffee, and around 80 mg/250 ml in commercial energy drinks. Caffeine tablets contain up to 200 mg/tablet [5, 122].

Pharmacodynamically, methylxanthines are adenosine receptor antagonists. The targeted A1 receptors display pre- and postsynaptic localization and mediate inhibitory effects. Thus, the disinhibition of adenosine receptors in the intralaminar mediothalamic thalamus improves cortical performance [5]. The inhibitory effect of methylxanthines on cAMP phosphodiesterase (PDE), however, plays only a minor role in the CNS at average plasma concentrations. Mobilization of Ca^2+^ from internal stores with an increase in [Ca^2+^]_i_, e.g., in the striatum and the reticular formation, has also been suggested.

Caffeine, the most important representative of “soft-enhancers,” exerts antagonistic effects on A1, A2A, A2B, and A3 receptors, inhibits IP3 receptors (IP3R1), and is an activator of ryanodine receptors (RyR1-3). Physiologically, a task-related increase in cerebral blood flow also seems to contribute to the caffeine effect [123].

The side effect profile of methylxanthines is dose- and user-dependent, including, i.a., agitation, irritability, tremor, angina pectoris, dysrhythmia, nausea, gastrointestinal complaints, lack of appetite, dyspepsia, and vomiting. Consequently, hypertension, hyperthyroidism, epilepsy, mania, schizophrenia, gastric, and duodenal ulcer are contraindications for methylxanthine ingestion [122, 124].

Meta-analyses of pivotal randomized controlled trials (RCTs) in healthy individuals revealed an increase in vigilance and attention, as well as—partly questionable—improvement in reaction rate and fatigue. However, a positive effect on memory performance and mood could not be proven [5]. Likewise, the subjective self-assessment does not seem to ameliorate [5]. It should be noted that caffeine is a prototypic ingredient of “soft-enhancers,” which are widely legitimated in society, e.g., for historical or traditional reasons.

### 6.2. Phenylethylamine Derivatives

Typical phenylethylamine derivatives include, i.a., dexamphetamine, lisdexamphetamine, amphetamine, methamphetamine, phenmetrazine, methylphenidate, fenethylline, pemoline, ephedrine, norephedrine, levopropylhexedrine, amfepramone, mefenorex, fenfluramine, and fenproporex. Pharmacodynamically, phenylethylamines act as modulators of the dopaminergic and noradrenergic system. Some promote the release of dopamine and norepinephrine from presynaptic nerve endings (so-called indirect dopaminergic and noradrenergic effects) and/or inhibit the reuptake of dopamine and norepinephrine into presynaptic nerve terminals [5, 125]. For some phenylethylamines, the molecular targets have been characterized in detail due to their great pharmacotherapeutic relevance. Methylphenidate, for example, serves as an inhibitor of the dopamine transporter (DAT, protein SLC6A3) and the noradrenaline transporter (NET, protein SLC6A2), which mediates the reuptake of both transmitters into presynaptic endings [126, 127]. Some phenylethylamines have been used as psychoanaleptics and anorectics. From a pharmacotherapeutic and regulatory perspective, the use of methylphenidate, dexamphetamine, and lisdexamphetamine is particularly relevant in the treatment of ADHD.

The side effect profile of phenylethylamines includes, i.a., euphoria, increased self-confidence, increased activity, reduction of fatigue and drowsiness, motor restlessness, and logorrhoea. Dysphoric states of anxiety and tension, as well as vegetative disorders, may also occur. However, we still lack reliable data concerning the long-term side effects of phenylethylamine ingestion [128]. Animal experiments, for example, provided evidence that high doses of methylphenidate could impair cerebral maturation [129].

In Germany, the prescribing rate for methylphenidate increased 10-fold from 1998 to 2008 (1998: 5 million defined daily doses (DDD); 2008: 53 million DDD) [130]. This steady increase up to 2012 has slightly declined since then, reaching 52 million DDD in 2017 [131]. By contrast, the prescribing rates for atomoxetine, the efficacy of which is estimated much lower than for methylphenidate, are stable at low levels (2017: 2 million DDD) [131]. Atomoxetine is largely irrelevant for pNE. Lisdexamphetamine, a prodrug of dexamphetamine with delayed-release kinetics for ADHD therapy, has experienced a strong annual increase in prescribing rates in Germany since its launch in 2013 (9 million DDD in 2017) [131]. The same holds true for dexamphetamine, which was approved in Germany for ADHD therapy in 2015 (0.48 million DDD in the year 2017, +51.7% compared to 2016) [131].

Although epidemiological studies suggest no augmented ADHD prevalence over the last decades, health insurance data indicate an increase in both diagnosed and treated ADHD patients over the last 25 years [132]. This increase seems to be largely attributable to adults with diagnosed ADHD, as the symptoms can persist in adulthood and old age [132, 133]. ADHD is a good example of the problem to distinguish properly between real diseases, and human behaviors or characteristics that we might happen to find disturbing [20, 134]. In the past 15 years, diagnoses of children with ADHD have also dramatically increased [135], as have prescriptions for drugs to control it. Critics argue that the diagnosis of ADHD is also about badly behaved children who cannot be controlled by parents and teachers. Proponents, on the contrary, state that children misbehave or act upon because they suffer from a disease that requires pharmacotherapeutic intervention [20]. As business literature illustrates, clinical diagnoses like ADHD are often well-accepted also as opportunities for market growth [136, 137]. In general, the thresholds for pharmacotherapeutic interventions tend to be lowered as becomes apparent, for example, in neuropsychiatric diseases (e.g., ADHD). The rigorous emphasis on complete physical well-being motivates individuals, whether they suffer from a defined disease or not, to try expensive and/or potentially harmful interventions. This might result in higher levels of medical dependency and risk and has a direct link to the use of pNE.

In Germany, the prescribing rate of psychostimulants in children and adolescents with ADHD (between 2009 and 2014) tends to decrease. However, in adults with persistent ADHD symptoms (approximately 25-60% of affected adolescents), the number is strongly increasing, particularly for methylphenidate-containing drugs [133, 138]. It is unclear, to which extend pNE related misuse of methylphenidate contributes to this phenomenon.

Undeniably, methylphenidate is one of the most commonly used psychostimulants for pNE [9, 33, 139, 140]. The percentage of non-ADHD individuals with alleged methylphenidate benefit is low [9, 141]. Non-ADHD diagnosed users of methylphenidate with a positive pNE-related experience typically exhibit high behavioral impulsiveness [9, 141] and are seven times more likely to be symptomatic for ADHD than control groups [59]. Psychostimulants such as methylphenidate are also used as self-medication in psychic alterations and sleep deprivation [142]. However, a clear distinction between a specific medical need for indicated treatment and (self-) medicated pNE is often sophisticated [3]. Moreover, the global pharmaceutical market for ADHD therapeutics is growing faster than expected, based on the rapidly expanding markets, mainly in the USA, Canada, and Australia [143].

The national diversities in epidemiology, drug regulation, and pharmacotherapeutic strategies in ADHD have raised concerns regarding the validity of ADHD diagnostics [144]. Differences include diagnostic thresholds, treatment expectations of clinicians, health insurers, teachers, parents, and treatment seekers [144, 145]. A recent systematic review revealed no evidence for a current increase in the number of children who strictly meet ADHD diagnostic criteria [146]. However, there are indisputable national specificities, e.g., in the USA, where ADHD is diagnosed more often as a condition requiring treatment in children (10.1% for ages 5 to 17 years, National Center for Health Statistics, 2015). In France, by contrast, the prevalence is only 3.5%-5.6% [147], and ADHD is treated predominantly psychosocially rather than pharmacologically [148]. Interestingly, in Iceland, methylphenidate prescription dramatically increased in 2010-2011 based on the increasing knowledge and awareness of ADHD in both the public and scientific/medical communities [44, 149, 150]. In addition, a high prevalence of intravenous methylphenidate abuse has also been reported, stressing the high abuse risk of methylphenidate in countries with increasing prescription rates [98]. National measures by the Directorate of Health of Iceland were taken to restrict prescription privileges to psychiatrists, neurologists, and pediatricians [98].

Country-specific differences might thus originate from specificities in national health care systems, drug licensing, and medical prescription [151–153] and accessibility to prescription psychostimulants [154]. Adderall™ (dexamphetamine, amphetamine), for example, is approved for the treatment of ADHD in the USA and is also prevalent among American students for pNE [154]. In most European countries, however, the drug is not authorized in this combination of active components because of its side-effect profile. While amphetamine improves attention and alertness in non-ADHD users, creativity is likely to be adversely affected [155]. As with other psychostimulants, these effects are highly dose-dependent [156].

RCT-based meta-analyses in healthy volunteers suggested that methylphenidate and dexamphetamine exert beneficial effects on attention and vigilance as well as on reaction rate. However, there was no clear improvement in memory performance [5, 6]. Mood and subjective self-assessments are also not positively affected, whereas high doses cause euphoria [5, 126].

### 6.3. Modafinil

Pharmacodynamically, modafinil is an atypical dopamine reuptake inhibitor of presynaptic DAT (dopamine transporter). In addition, modafinil was reported to serve as an inhibitor of the norepinephrine transporter (NET) [157]. Furthermore, modafinil modulates GABAergic and glutamatergic neurotransmission. Collectively, modulating all of these targets finally promotes attention and vigilance. Therapeutic indications for modafinil are narcolepsy, chronic shift worker syndrome, and sleep apnea syndrome with excessive daytime sleepiness (when other measures like continuous positive airway pressure (CPAP) are inappropriate) [158]. The side effects include, i.a., tachycardia, hypertension, palpitation, tremor, restlessness, headache, dizziness, dry mouth, gastrointestinal disorders (vomiting, nausea, diarrhea), visual impairment, drowsiness, insomnia, Stevens-Johnson syndrome (SJS), toxic epidermal necrolysis (TEN), and Drug Rash with Eosinophilia and Systemic Symptoms (DRESS) [159, 160].

While modafinil is rarely used as a cognitive neuroenhancer in German-speaking countries, [30, 139] modafinil is the most widely used neuroenhancer in the UK [140]. RCTs in healthy volunteers suggest a eugeroic effect with increased attention and vigilance, reduction in response time, and fatigue. Evidence for positive cognitive effects in healthy individuals has been reported [161, 162]. Also, modafinil seems to affect mood and emotional processing positively [5, 91, 92, 163–165].

### 6.4. Cocaine

Pharmacodynamically, cocaine acts via inhibition of dopamine and noradrenaline reuptake on DAT and NET in presynaptic nerve terminals. Antagonistic effects on the serotonin receptors 5-HT3A and 5-HT3AB have also been described. Cocaine uptake increases physical performance and causes psychomotor stimulation and euphoria. Due to its strong addictive potential, cocaine is not therapeutically relevant.

### 6.5. Nootropics (Antidementia Drugs, Psychoenergetics)

Pharmacodynamically, nootropics such as donepezil, tacrine, and rivastigmine act as acetylcholinesterase (AChE) inhibitors. In addition, tacrine and rivastigmine also serve as butyrylcholinesterase (BChE) inhibitors and mediate increased ACh levels at central synapses. Furthermore, tacrine is an allosteric modulator of muscarinic M1 and M2 ACh-receptors. In addition to AChE inhibition, galantamine leads to allosteric modulation of the nicotinic ACh-receptor (AChN-R) with enhanced endogenous ACh-activity. Nootropics are typically used in moderate Alzheimer's disease and mild to moderate dementia associated with Parkinson's disease. The side-effect profile of this group includes substance-specific gastrointestinal disorders (nausea, vomiting, diarrhea), headache, tremor, loss of appetite, urinary incontinence, dose-related hallucinations, agitation, and aggressiveness.

Other important nootropics are piracetam, pyritinol, vinpocetine, and deanol. Piracetam is a positive allosteric modulator of GluA1, GluA2, GluA3, and GluA4 receptors (ionotropic glutamate receptors). Pyritinol acts as a pyridoxine (vitamin B6) derivative. It influences cerebral metabolism, as well as glucose uptake and protein metabolism, promotes stabilization of cell membranes, increases ACh-synthesis and ACh-release, and improves microcirculation in the CNS.

Vinpocetine, a derivative of the vinca alkaloid vincamine, is a PDE 1A and 1C inhibitor. It aims to improve cerebral blood flow and mediate neuroprotective effects via inhibition of voltage-gated Ca^2+^ channels (Ca_v_).

Recently, memantine has gained special attention as psychoenergetic. Memantine is a noncompetitive, partial NMDA-receptor antagonist for the treatment of moderate to severe Alzheimer's disease. Adverse reactions include hypertension, headache, dizziness, somnolence, constipation, and in overdosed amounts, fatigue, drowsiness, weakness, agitation, and gastrointestinal disorders. The prescribing rates for cholinesterase inhibitors are highest among antidementia drugs and have been rising almost linearly since 2005. This also holds true for memantine. However, the prescribing rates have been relatively stable since 2014 (2017: 34 million DDD in Germany). The dominant cholinesterase inhibitors in Germany (2017: 63 million DDD) are donepezil (2017: 36 million DDD, +9.3% compared to 2016), rivastigmine (2017: 13.9 million DDD, -1.2% compared to 2016), and galantamine (2017: 7.5 million DDD, +0.8% compared to 2016) [131]. Prescribing rates for piracetam are decreasing (2017: 7 million DDD) [131]; for nicergoline, however, there is an increase (2017: 1 million DDD) [131]. In general, prescription rules and regulatory frameworks affect these developments in prescription rates [166]. In Germany, for example, the decrease in piracetam prescription rate was related to a Cochrane review that stated that the efficacy of piracetam in the treatment of dementia and cognitive deficits was not supported by literature [167]. In addition, the German S3 guideline for dementia did not recommend piracetam treatment due to a lack of evidence of efficacy.

For donepezil, rivastigmine, and memantine, RCT-based meta-analyses in healthy individuals did not reveal clear evidence of positive effects on attention, vigilance, response time, fatigue, and memory [5, 92, 168]. For donepezil, deterioration of memory function in elderly volunteers has been described [169].

### 6.6. Ginkgo Biloba

The standardized ginkgo biloba extract EGb761 contains approximately 22-27% flavone glycosides; 5-7% terpene lactones such as the diterpenes ginkgolide A, B, C, J, and M; sesquiterpene bilobalide; and several other components [170]. Pharmacodynamically, the ginkgo biloba extract was supposed to serve as a polyvalent free radical scavenger [171], to enhance microperfusion, to reduce blood viscosity by inhibition of platelet-activating factor [170], to improve cholinergic, dopaminergic, and glutamatergic neurotransmission [172], and to attenuate *β*-amyloid aggregation [173]. The prescribing rates for ginkgo biloba extracts are stable since 2008 and remain at a much lower level compared to nootropics [131]. The efficacy of ginkgo biloba extracts in patients with dementia or mild cognitive impairment is still a question of debate. A 2009 Cochrane analysis did not see any clear evidence of a clinical benefit for patients suffering from AD [170, 174, 175]. However, recent meta-analyses suggest positive effects of gingko biloba extracts in dementia with only minor side-effect profile [176, 177]. Meta-analyses of pivotal RCTs in healthy volunteers revealed potential positive effects in attention and vigilance, response rate, fatigue, and memory, however, to a lesser extent.

### 6.7. Alcohol and Benzodiazepines

Alcohol and benzodiazepines play a minor role in pNE (indirect pNE) as they can reduce anxiety and tension. The GDS 2017 revealed the highest prevalence for alcohol in Hungary (29.4%), France (22.8%), Canada (22.7%), Brazil (21.4%), and the USA (20.9%). Germany displayed a lower rate of 10.3%. For anxiolytic benzodiazepines, German-speaking countries exhibit low prevalences (Germany 1.9%, Austria 2.7%, Switzerland 2.9%), while Brazil and the USA dominate with 11.5% and 14.0%, respectively [50, 97].

### 6.8. Beta-Adrenoceptor Antagonists

Beta-blockers, which among others can mitigate the somatic concomitants of stress and tension, are slightly represented among pNE. In Germany, according to GDS 2017, 0.5% of the participants make use of them. In Brazil (2.0%), the US (2.0%) and Portugal (3.3%), the numbers are higher [50, 97].

### 6.9. Cannabis

Similar to alcohol, benzodiazepines, and beta-adrenoceptor antagonists, cannabis and its ingredients (cannabinoids) are used for indirect cognitive pNE. They are supposed to cause a reduction in nervousness and anxiety and to improve relaxation and performance on the following day [2, 30, 50, 95, 139]. According to the GDS 2017, cannabis intake for indirect pNE is highest in the USA (52.5%) and Canada (42.5%), lowest in New Zealand (14.6%), Australia (17.4%), and in German-speaking countries, i.e., Germany (11.0%), Austria (17.6%), and Switzerland (15.1%) [50, 97]. In the latter, restrictions in availability and accessibility of cannabinoids confine the use of cannabis, also for indirect pNE.

### 6.10. Other Potential Enhancers

Two attractive candidates include the neuropeptide Cerebrolysin® [178] and Montelukast, which is a leukotriene receptor antagonist for the treatment of bronchial asthma. In animal experiments, montelukast induced a significant enhancement in learning and memory as well as increased neurogenesis in older animals [179]. Besides, other “soft enhancers” such as dietary supplements/phytotherapeutics are gaining increasing attention by pNE users. Although vitamins (e.g., Vit. A, Vit. B complex, Vit. E) are mandatory for proper development and function of the CNS and vitamin deficiencies can result in devastating disorders, excessive ingestion beyond the recommended DDD does not increase cognitive performance in healthy individuals [180].

### 6.11. Antidepressants: Mood Enhancers

Current pharmaceuticals used for mood enhancement include, i.a., fluoxetine, citalopram, dapoxetine, escitalopram, fluvoxamine, paroxetine, sertraline, and venlafaxine. Pharmacodynamically, relevant subgroups contain the selective serotonin reuptake inhibitors (SSRIs), selective norepinephrine reuptake inhibitors (SNRIs), and selective serotonin-norepinephrine reuptake inhibitors (SSNRIs). The main side effects of these antidepressants include gastrointestinal disorders (nausea, vomiting, diarrhea, constipation), nervousness, insomnia, and drowsiness. Overdose may cause a serotonergic syndrome with tachycardia, hypertension, nausea, vomiting, diarrhea, restlessness, hallucinations, tremor, hyperreflexia, and seizures.

Notably, prescribing rates for antidepressants are steadily increasing, with 1,491 million DDD in 2017 in Germany. This development is mainly based on SSRI and SNRI prescribing rates, with 677 million DDD and 280 million DDD, respectively, for 2017. Prescribing rates for tricyclic antidepressants (TCAs), on the other hand, are slowly decreasing with 248 million DDD in 2017 [131].

In general, prescribing rates can be substance and region-specific and are affected, i.a., by regional traditions, marketing strategies, but even more important by regulatory means. For TCAs, for example, it turned out that they are potentially inappropriate medications in the elderly and several guidelines do not recommend TCA use in the elderly. Based on the Beers criteria [181, 182], PRISCUS list [183], Norwegian General Practice (NORGEP) criteria [184], and French consensus panel list [185], TCAs are potentially inappropriate medications for the elderly regardless of the disease. Similar official suggestions were made in South Korea [186]. In Canada, on the other hand, TCA prescription increased (in children) from 2012 to 2016. Consistent with the Canadian data, from 2005 to 2012 antidepressant prescriptions for young patients have increased from 2005 to 2012 in the United States, the United Kingdom, Denmark, Germany, and the Netherlands [187, 188]. There have also been recent reports of increasing prescription rates in the United Kingdom and Australia [189–191]. These and other examples, e.g., from SSRIs [192], illustrate how official recommendations from professional health committees and regulatory bodies can influence prescription rates.

In the context of neurorehabilitation, antidepressants were reported to exert positive effects on neuroplasticity [193]. However, there is also evidence that SSRIs adversely affect neural connectivity between cortical and subcortical areas [194]. The predominant motivation for healthy individuals to ingest antidepressants is to improve mood and social interaction/social skills, to reduce or abolish social anxiety, and to eliminate insecure behavior. RCT-based meta-analyses in healthy volunteers, however, did not prove any positive effects on mental performance/cognition. In contrast, there are reports that some antidepressants might impair cognition. Importantly, no positive effects on mood became evident in healthy individuals [5, 92].

## 7. Experimental Approaches and Drug Development: Novel Potential Cognitive Enhancers at the Prelicensure Stage

Examples of experimental cognitive enhancers are rolipram, D-cycloserine, and ampakine. Pharmacodynamically, rolipram acts via inhibition of PDE 4A, 4B, 4C, and 4D, leading to an increase in cytosolic cAMP and to enhanced CREB activity. D-cycloserine serves as a partial agonist at NMDA-receptors associated with LTP enhancement and anxiety extinction. Efficacy was demonstrated in acrophobia and studies on exposure-based psychotherapy [91, 195]. Ampakines are positive allosteric modulators of AMPA- (non-NMDA) receptors. They increase glutamatergic activity in the CNS and mediate an increase in growth factor levels (e.g., BDNF). One representative is aniracetam, which exerts anxiolytic effects by interaction with D2 receptors, nicotinic acetylcholine receptors (AChN-R), and 5-HT2A receptors. Furthermore, aniracetam causes an allosteric potentiation of AMPA-receptor (GluA1-4 receptor) activation.

Multiple, potentially innovative pharmaceutical approaches in treating dementia have failed in recent years. Nevertheless, the development of new antidementia drugs in the future might aggravate the potential misuse of pNE. A survey of clinicaltrials.gov revealed that as of January 30, 2018, a total of 112 new drugs for the treatment of Alzheimer's dementia were in phase I-III of clinical trials. 63% were so-called disease-modifying drugs, 12% were used for the treatment of neuropsychiatric symptoms associated with dementia and behavioral disorders, and 22% were classified as symptomatic cognitive enhancers [196].

Biosynthetic recombinant human insulin (rHI) for intranasal administration is being tested in phase III and is supposed to increase cellular signaling and neurogenesis at the metabolic level to enhance cognition. Another cognitive enhancer in phase III is octohydroaminoacridine, a new AChE inhibitor. Other novel phase III components target Sigma- and NMDA-receptors, the serotonergic, noradrenergic, and orexin systems. The latter are supposed to improve neuropsychiatric symptoms, such as agitation and sleep disorders [196].

Twenty-one potential cognitive enhancers are listed in phase II clinical trials. They affect the sigma and muscarinic receptor system as well as glycine transporters. Other novel components include modulators of protein kinases (PKC, tyrosine kinase Fyn), PDE 3 antagonists, AChE inhibitors, NMDA modulators, and modulators of neurogenic peptide hormones, growth factors, and oxidative stress [196].

Phase I studies include four cognitive enhancers, which serve as GABA modulators, AChE- and BChE-inhibitors, and muscarinic and serotonin receptor modulators [196]. It remains to be determined how many antidementia drugs with potential cognitive enhancement character will eventually be licensed and marketed [196, 197]. Another recent update on novel cognitive enhancers was provided by Napoletano et al. (2020) [198].

Undoubtedly, drug research and development in dementia will increase the repertoire of potentially available drugs for pNE in healthy individuals in the future.

## 8. Ethical Aspects of pNE

An all-encompassing discussion of ethical aspects in pNE is clearly beyond the scope of this work. However, it seems justified to pinpoint some central ethical aspects that can be subject to further discussion.

The numerous advantages of pNE for an individual are apparent: increase in cognitive performance, sleep reduction, mood enhancement, improvement of executive functions, reduction of psychosomatic side effects, increase in efficiency and controllability of mental processes, hedonism, an individual complication-free adaptation to external and internal requirements, and no “hardships of learning” [83, 199, 200]. Further advantages might include benefits for the society due to reduced resting periods, greater competitiveness, increased productivity and efficiency, advantages on the job market, and related higher living standards [83, 201]. The economic and social benefits could be dependent on reliable, consistent, and sustainable cognitive performance, emotional stability, and intellectual creativity and innovation [83].

In contrast, there are numerous, partially fatal disadvantages of pNE, such as negative consequences on individual and public somatic and mental health, furthermore the dissolving of a clear distinction between work and life undermining work-life balance. Uncontrolled competition among students, academics, and other employees, related physical and mental health implications; globalization of social life; and a consumer-focused society with disintegration of traditional moral conceptions are other major concerns [83].

The futuristic horror scenario of an “enhanced society” causes several dilemmas, which have been addressed in part, in the sections above, but which are elaborated here in a broader context.

The apparent ethical dilemma seems to be a theoretical one at the first glance. It directly affects the philosophical concepts of brain-mind relation [83, 202, 203]. From a neuroethical point of view, the exceptional ethical implications of pharmacological brain modulation by pNE have to be considered, in specific, the questioning of human subject quality and its authenticity [200]. Pharmacological neuroenhancement can change personality and human image. In addition, the rapidly increasing qualitative and quantitative intellectual requirements of modern society which we are facing today are beyond our original evolutionary adaptation [204].

In contrast to this basic philosophical discussion, there are more concrete ethical implications. One is the social dilemma originating from pNE use. There is hardly any doubt that pNE can deteriorate the meaningfulness of effort. It can create unfair competition and do substantial harm to the community and solidarity, promote social injustices, and cause group dynamic constraints. In general, pNE is classified as “fraud,” particularly if it is not available to everyone [200]. Some authors have thus specifically focused on the question of drug distribution (availability/accessibility, see Section 5) and initiated a discussion about making pNE drugs freely available upon request [42] and legalize the pNE market.

As outlined above, there are currently numerous, predominantly illicit ways to acquire pNE drugs, e.g., via local distribution or IOPs. Importantly, it is highly questionable whether pNE drugs obtained from friends/dealers or IOPs meet the quality criteria of originally prescribed medication, and thus, the black market per se poses a tremendous risk to the health of individuals and public health in general. It might be tempting to speculate that legalization of the pNE market could reduce or eliminate a black market and be beneficial for safety aspects of pNE use. However, this potential positive effect of legalization seems to be shortsighted for several reasons. Practically, it is illusionary that there will be a worldwide agreement in the legalization of the pNE market. There are fundamental country and region-specific differences in tradition, attitude, and philosophy regarding the aspect of neuroenhancement and its pharmacological modulation. Furthermore, there are still regulatory/legal differences as well, although there are attempts of harmonization in general. Enhancing accessibility by legalization of the market would be like the bursting of a dam with uncontrolled use and without consultation of a physician who could inform the potential user about adverse effects and contraindications. In addition, there would be no systematic monitoring of potentially harmful long-term effects of pNE drug use, and we are still lacking the most relevant information. Another concern is the interaction with other medications which might have devastating consequences for patients; particularly, as pNE is normally performed without supervision or advice by a physician. As a worst-case scenario—with users suffering from preexisting conditions—short- and long-term adverse effects of pNE use could result in acute life-threatening conditions or exert chronic somatic and/or mental damage. Based on our knowledge today, we are clearly lacking substantial information of long-term effects of pNE use on cognition, affection, and the somatic field in healthy individuals.

Importantly, regulatory restrictions and recommendations from professional health committees have the power to influence the use of pNE drugs as shown in Section 6. Furthermore, countries with restrictive handling of pNE drugs exhibit lower prevalences than those with less restrictive regulations (see Section 4).

It should also be considered that free accessibility to pNE drugs might enhance a shift from using “soft” neuroenhancers to “hard” neuroenhancers. Currently, many potential users still fear the risks of prescription pNE drugs and focus on, e.g., methylxanthines containing products or appropriate physical activities, meditation, and yoga. Nowadays, they might still try to change their lifestyle by gaining sufficient amounts of rest and sleep, improve the quality of nutrition, or use computing devices or brain training software [83, 205]. Alleviation of accessibility would likely enhance the usage of “hard” enhancers.

However, a high percentage of students, academics, and other employees does not want to use pNE drugs, but they might feel forced to take them in order to successfully compete with pNE users, particularly in their professional life. This kind of social dilemma generates a societal conformity pressure and potential avoidance strategies and has substantial implications on the individual freedom of decision-making.

A special, not self-related ethical issue of pNE is related to its altruistic use by, e.g., physicians, surgeons, nurses, caretakers, soldiers, pilots, flight controllers, police officers, and firefighters in national or global emergencies/catastrophes. This aspect became urgent these days in the worldwide COVID-19 (Coronavirus SARS-CoV-2) pandemic. Even under such circumstances, however, it must be clear to executives, persons in charge, and involved professionals that pNE can pose severe health risks upon use.

The different views on ethics and related medical, legal, and social issues have been discussed controversially in the past, and the ethical aspects and dilemmas associated with pNE in healthy individuals are subject of an ongoing debate [83].

## 9. Conclusions

Although pNE can serve the restitution of physical, mental, and social health under specific disease-related circumstances, its use in healthy individuals does not serve these criteria. The fact that our current sociocultural environment forces people to use pNE to cope with the physical, mental, and social implications in a rapidly changing digital world, it suggests that our current work-life environment is pathogenic. Each individual is characterized by her/his specific physiological, physical, and mental capabilities. pNE shifts these physiological conditions to overcome individual restrictions in an unphysiological/superphysiological manner. Notably, the consequences of “physiology at its limits” on the somatic and mental level remain unknown. One necessary consequence would be to redefine and adapt our work-life philosophy and to provide and implement powerful and effective tools of resilience systematically. Clearly, with the exception of emergency situations, pNE is not an option in healthy individuals, considering the potential side effects of drug intake, sociocultural consequences, and ethical implications. Again, from a drug regulatory point of view, the benefit/risk is always negative as the risks, i.e., the potential side effects always outweigh the benefit in this case, particularly as side effects might cause disease states that need further medical attention and treatment. pNE for the sake of optimized skills to meet excessive social, educational, or economic demands should step back behind the physical and mental integrity of the individual. This should be the highest maxim.

Current RCTs suggest that only methylphenidate, amphetamines, and modafinil exert a verifiable positive effect on attention and vigilance in healthy individuals. However, improvements in memory function and mood—as anticipated by users—cannot be verified by meta-analyses in healthy volunteers. Neuropsychological comparative studies with caffeine, dexamphetamine, and modafinil in young, healthy individuals reveal no significant differences in attention and vigilance [206, 207]. Considering that socially “legitimated enhancers,” e.g., caffeinated drinks produce comparable effects on attention and vigilance, the latter exhibit a superior “benefit/risk” ratio. However, this may change in the future with the development of new nootropics.

Recent pharmacoepidemiological studies demonstrate that pNE is increasing globally. In the USA/UK, the overall prevalence is the highest. In Germany, the prevalence is still low, but also increasing. Country-specific sociocultural aspects and peculiarities of national health care systems have a central effect on this development. In Germany, the high level of education among applicants, the critical attitude towards the risks of neuroenhancers, and restrictions in availability/accessibility contribute to the rather low prevalence. Nevertheless, a significant proportion of pNE applicants obtains neuroenhancers via medical prescription, which undermines regulatory restrictions. Adhering to regulatory rules is one way to limit an uncontrolled spread of pNE.

An important question is whether societally, academically, or legally prescribed values and theoretical positions on pNE are also reflected in lay people's empirical understandings and perceptions of mind doping. Given the interrelation of pNE and health definition outlined above, it seems mandatory that health professionals, politicians, and other stakeholders know what the people perceive as the most relevant aspects regarding health, in particular, what health is, and which factors in people's lives constitute health. A continuing ethical and social debate, pharmacoepidemiologic and pharmacovigilance studies, integration of expert knowledge, and international regulations are urgently needed in order to prevent pNE from escalating globally.

## Figures and Tables

**Table 1:** Neuroenhancement and mood enhancement—substance classes and pharmacodynamics.

Substance class	(https://pubchem.ncbi.nlm.nih.gov/search/search.cgi)	Exemplary active components	Pharmacodynamical targets	Effects for pNE in healthy individuals (from RCTs, see Section 6)
Methylxanthine derivatives	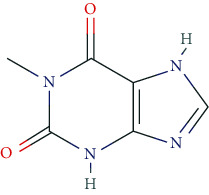 7-Methylxanthine	Caffeine Theophylline Theobromine	Adenosine receptor antagonist (A1, A2A, A2B, A3) IP3R1 antagonist RyR1-3 activator cAMP-PDE-inhibitor Ca^2+^-homeostasis modulator	Improvement of attention, but not memory and mood
Phenylethylamine derivatives	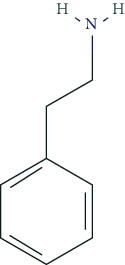 Phenethylamine	Methylphenidate Dexamphetamine Lisdexamphetamine Methamphetamine Fenetylline Pemoline Ephedrine Norephedrine Levopropylhexedrine Amfepramone Mefenorex Fenfluramine Fenproporex	DAT and NET inhibitor Facilitation of dopamine and norepinephrine release	Improvement of attention, but not memory Euphoria in high dose
Modafinil	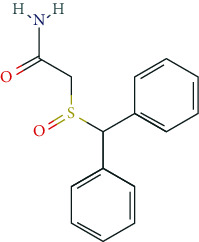		DAT and NET inhibitor Modulator of the GABAergic and glutamatergic neurotransmission system	Improvement of attention, cognition, and mood
Cocaine	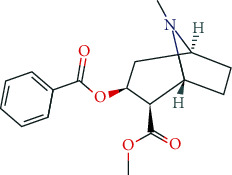		DAT and NET inhibitor Serotonin receptor antagonist (5-HT3A, 5-HT3AB) Modulator of the GABAergic and glutamatergic system	Psychomotor stimulation and euphoria, strongly addictive
Nootropics (antidementia drugs, psychoenergetics)	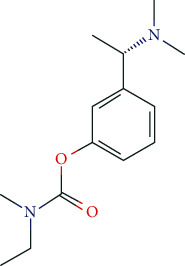	Rivastigmine	AChE and BChE inhibitors	No clear evidence of improvement of attention and memory
	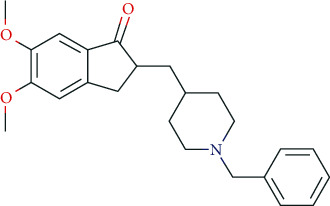	Donepezil	AChE inhibitor M1 and M2 receptor antagonist	
	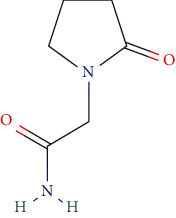	Piracetam	GluA1, GluA2, GluA3, GluA4 receptor modulator	
	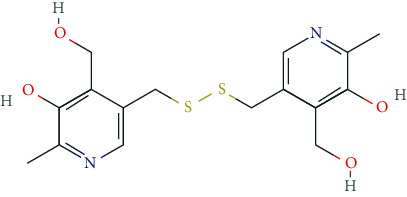	Pyritinol	Pyridoxin (vitamin B6) derivate	
	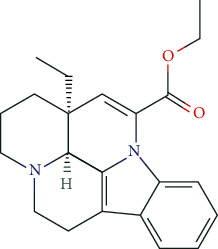	Vinpocetine	PDE1A and PDE1C inhibitor VGCC blocker	
	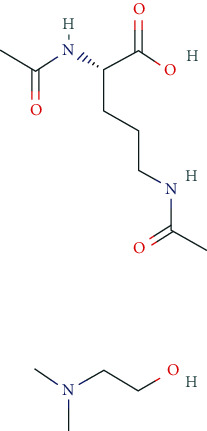	Deanol	Precursor of ACh synthesis	
	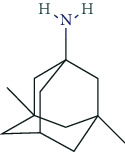	Memantine	Non-competitive partial NMDA receptor antagonist	
Ginkgo biloba	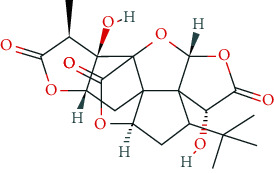 e.g., ginkgolide B	Flavone glycosides Terpene lactones Sesquiterpene Bilobalide	Radical scavenger Improvement of cholinergic, dopaminergic and glutamatergic neurotransmission	Improvement of attention and memory under discussion
Benzodiazepines	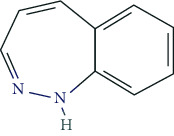 Benzodiazepine	Bromazepam Diazepam Lorazepam	Interaction with GABAergic system	Reduction of anxiety and tension
Alcohol	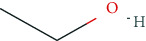 Ethanol	Ethanol	Interaction with GABAergic, opioid, serotonergic, glutamatergic, and dopaminergic systems	Reduction of anxiety and tension
Beta-adrenoceptor antagonists	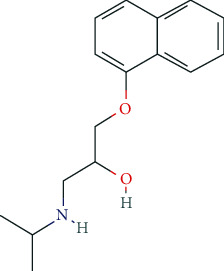 e.g., propanolol	Propranolol Atenolol Carvedilol	Blocker of beta-adrenoceptors	Reduction of stress and tension
Cannabis	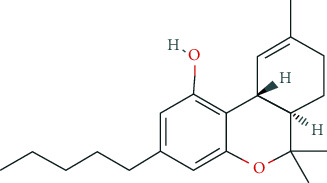 e.g., tetrahydrocannabinol	Tetrahydrocannabinol Cannabidiol	Interaction with cannabinoid receptors	Reduction of reduce anxiety
Other potential enhancers		Cerebrolysin® (neuropeptides)	Modulation of neurotransmitter systems (e. g., glutamatergic, cholinergic, serotonergic, and dopaminergic) Cotransmitter	
	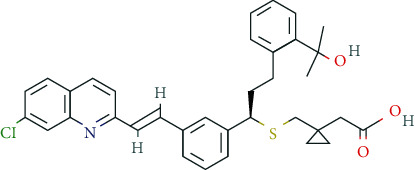	Montelukast	Leukotriene receptor antagonist	Potential improvement of learning and memory
	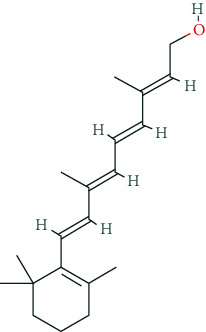 e.g., vitamin A	Vitamins	Cofactors and coenzymes	No cognitive improvement
Antidepressants—mood enhancers	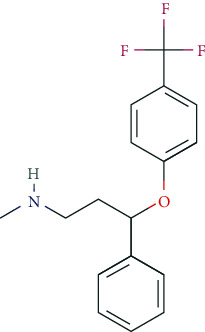 e.g., fluoxetine	Fluoxetine Citalopram Dapoxetine Escitalopram Fluvoxamine Paroxetine Sertraline	Selective serotonin and/or norepinephrine reuptake inhibitors (SSRI, SNRI, SSNRI)	No clear evidence of cognitive or mood enhancement

## Data Availability

This is a review article and thus a data availability statement is not applicable.
